# Efficacy and safety of Chinese herbal medicine Danggui Sini decoction for knee osteoarthritis: A protocol for systematic review and meta-analysis

**DOI:** 10.1097/MD.0000000000031516

**Published:** 2022-11-18

**Authors:** Xing Zhou, Ke-Meng Xiang, Jinlei Li, Guang Yang, Yanbo Wang, Hanting Xia, Rujie Zhuang

**Affiliations:** a The First Clinical College, Zhejiang Chinese Medical University, Zhejiang, China; b Department of Orthopaedics, The First Affiliated Hospital of Zhejiang Chinese Medical University, Zhejiang, China; c Taizhou Traditional Chinese Medicine Hospital, Zhejiang, China; d Kunming Traditional Chinese Medicine Hospital, Yunnan, China.

**Keywords:** Danggui Sini decoction, knee osteoarthritis, protocol

## Abstract

Knee osteoarthritis (KOA) often causes joint pain, weakness and mobility disorders, which seriously affects people’s daily life and makes them unable to work and study normally. Traditional Chinese medicine (TCM) prescription Danggui Sini Decoction (DGSND) has been widely used in clinical practice and achieved good results. But there is no high-level evidence to support this result. The aim of this study is to evaluate DGSND’s efficacy and safety in the management of KOA.

**Methods:**

We will search 7 electronic databases including Chinese National Knowledge Infrastructure (CNKI), Wanfang Data (WF), Chinese Scientific Journals Database (VIP), Chinese databases SinoMed (CBM), PubMed, Embase, and Cochrane Library databases. All the publications, with no time restrictions, will be searched without any restriction of language and status, the time from the establishment of the database to September 2022. Two reviewers will independently assess the quality of the selected studies, NoteExpress and Excel software will be used to extract data, and the content will be stored in an electronic chart. Different researchers will separately screen the titles and abstracts of records acquired potential eligibility which comes from the electronic databases. Full-text screening and data extraction will be conducted afterward independently. Statistical analysis will be conducted using RevMan 5.4 software.

**Results:**

This study will compare the effects of DGSND and any other different methods on patients with KOA to provide high-quality, evidence-based clinical recommendations.

**Conclusion:**

The study provides a trustable clinical foundation for DGSND in the treatment of KOA.

## 1. Introduction

Knee osteoarthritis (KOA) is a common degenerative disease, with a higher proportion of the middle-aged and elderly people.^[1]^ As the age increases, the prevalence rate of KOA is increasing, to reach 15.0% in people aged 85 years and older.^[2]^ Although most patients with KOA suffer from knee joint swelling, deformity, knee extensor muscle weakness, and pain, the main demand of this group of people is to solve the pain and dysfunction and restore a higher quality of life as soon as possible.^[3–5]^ In the treatment and management of symptomatic KOA, oral nonsteroidal anti-inflammatory drugs (NSAIDs) and intra-articular injections of drugs including PRP, sodium hyaluronate, and hormones have achieved good clinical effects. However, long-term use of NSAIDs will increase the incidence of gastrointestinal and cardiovascular adverse events, and invasive procedures may also bring the risk of joint infections.^[6–8]^ Noninvasive conservative treatment can also be beneficial.^[9]^ Traditional Chinese medicine (TCM) occupies a relatively important position among them. An increasing number of people tend to use TCM to improve their symptoms.^[10,11]^

Chinese herbal medicine has been widely used in the clinical treatment of various diseases. It has a good compatibility and dosage based on the theory of TCM syndrome differentiation, and it has good effects and few adverse reactions.^[12]^ Danggui Sini decoction (DGSND) was recorded in “Shanghanlun Treatise on Febrile Diseases” which is a classic Chinese medicine book. It is composed of 7 TCMs: the roots of Radix Angelicae sinensis (Dang-Gui), branches of Cinnamomum cassia Presl (Gui-Zhi), the roots of Paeonia lactiflora Pall (Bai-Shao), the whole herb Asarum heterotropoides (Xi-Xin), the stem of Tetrapanax papyriferus (Tong-Cao), the roots of Glycyrrhiza uralensis (Gan-Cao), and the fruits of Ziziphus jujuba Mill (Da-Zao).^[13]^ Studies have shown that DGSND can establish cross-talk with many downstream inflammatory pathways, exert anti-inflammatory effects, and regulate the proliferation and apoptosis of chondrocytes by intervening in the PI3K-Akt signaling pathway. At the same time, HIF-1 expression was used to ensure the normal function and metabolism of knee joint under hypoxia condition, this feature of multiple targets and multiple pathways play an important role in the treatment of KOA.^[14,15]^

Currently, many scholars firmly believe that DGSND can relieve and improve the symptoms of KOA patients, but there is no higher-level evidence-based medical evidence to systematically evaluate and analyze the safety and efficacy of DGSND in the treatment of KOA. Therefore, this study aims to achieve the above-mentioned goals through systematic reviews and meta-analysis, and provide reliable evidence for the clinic.

## 2. Methods

### 2.1. Study registration

This protocol report is structured according to the Preferred Reporting Items for Systematic Reviews and Meta-analysis Protocols (PRISMA-P) statement.^[16]^ It is registered on the International Prospective Register of Systematic Reviews. (Registration number: CRD42022361901).

### 2.2. Inclusion criteria

#### 2.2.1. Type of study.

Only randomized controlled trials (RCTs) will be included irrespective of blinding, publication status, or language in this study.

#### 2.2.2. Types of participants.

Patients were diagnosed with KOA and the study belongs to a randomized controlled trial. Clinical results included VAS, clinical effectiveness and the incidence of adverse reaction. The experimental group must contain DGSND or modified DGSND and the control group was not limited except that. Otherwise, studies will be excluded if they cannot meet the inclusion criteria.

#### 2.2.3. Types of interventions.

Interventions of the experimental group are DGSND or modified DGSND. There are no restrictions on the way of administration, dosage, and treatment period.

#### 2.2.4. Types of control groups.

The control group has other treatment methods different from DGSND or modified DGSND.

#### 2.2.5. Outcomes.

The outcomes will include VAS,^[17]^ clinical effectiveness, the incidence of adverse reaction.

### 2.3. Search strategy

CNKI, Wanfang, VIP, CBM, PubMed, Embase, and Cochrane Library databases were searched for this study. Take the subject terms combined with free words to search, take PubMed as an example: terms consist of disease (arthritis OR osteoarthritis, knee OR knee osteoarthritis OR knee osteoarthritides OR Osteoarthritis of Knee OR Osteoarthritis of the Knee) and intervention (Danggui Sini decoction OR Danggui Sini Tang OR modified Danggui Sini decoction) and research types (randomized controlled trial OR controlled clinical trial OR random trials). The searches will be conducted by two authors independently (ZX and XKM) as shown in Table 1.

**Table 1:** Pubmed database search strategy.

No	Search items
#1	“ osteoarthritis, knee” [Mesh]
#2	osteoarthritis, knee [Title/Abstract]
#3	arthritis [Title/Abstract]
#4	knee osteoarthritis [Title/Abstract]
#5	knee osteoarthritides [Title/Abstract]
#6	Osteoarthritis of Knee [Title/Abstract]
#7	Osteoarthritis of the Knee [Title/Abstract]
#8	1 OR 2 OR 3 OR 4 OR 5 OR 6 OR 7
#9	Danggui Sini decoction [Title/Abstract]
#10	Danggui Sini Tang [Title/Abstract]
#11	modified Danggui Sini decoction [Title/Abstract]
#12	9 OR 10 OR 11
#13	randomized controlled trial [Title/Abstract]
#14	controlled clinical trial [Title/Abstract]
#15	random trials [Title/Abstract]
#16	13 OR 14 OR 15
#17	8 AND 12 AND 16

### 2.4. Data collection and analysis

#### 2.4.1. Selection of studies.

**Figure 1.**
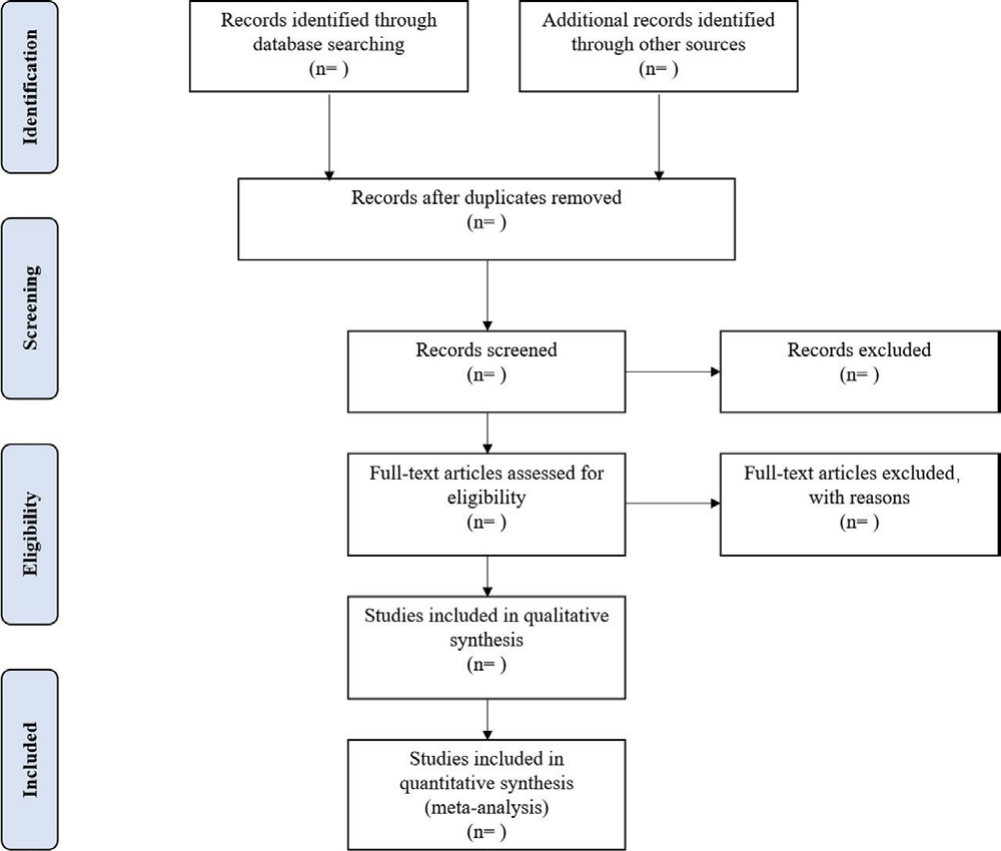
Flowchart of literature selection.

Different researchers (ZX and YG) will separately screen the titles and abstracts of records acquired potential eligibility which comes from the electronic databases. The obtained literature is managed by NoteExpress, irrelevant and duplicate articles are excluded by reading the title and abstract. Full texts screening and data extraction will be conducted afterward independently, and finally selected according to the inclusion criteria. Any disagreement will be resolved by discussion with the third author (LJL) until consensus is reached or by consulting a third author. PRISMA-P flowchart will be used to show the selection procedure (Fig. 1).

#### 2.4.2. Data extraction and management.

NoteExpress and Excel software will be used to extract data, and the content will be stored in an electronic chart. The following data will be extracted: author, year of publication, country, interventions of experimental groups and control groups, time point, outcome measures, age of patients, the total number of people included in the study, patients’ basic information, etc. Different researchers will separately extract data. Any disagreement regarding data extraction will be resolved by discussion until consensus is reached or by consulting a third author.

### 2.5. Risk of bias assessment

Two reviewers (ZX and XKM) will independently assess the quality of the selected studies according to the Cochrane Collaboration’s tool for RCTs.^[18]^ Items will be evaluated in three categories: low risk of bias, unclear bias, and high risk of bias. The following characteristics will be evaluated: random sequence generation (selection bias), allocation concealment (selection bias), blinding of participants and personnel (performance bias), incomplete outcome data (attrition bias), selective reporting (reporting bias), and other biases. Results from these questions will be graphed and assessed using Review Manager 5.4. The results will be presented in the form of a graph and will be independently evaluated by two researchers (ZX and XKM). If there are differences of opinion, they will be discussed with the third researcher (LJL).

### 2.6. Statistical analysis

Statistical analysis will be conducted using RevMan 5.4 software (Cochrane Collaboration, London, UK). For continuous data, will be used mean difference (MD) as the effect indicator with 95% confidence interval, and dichotomous data will be calculated as risk ratio (RR) or odds ratio (OR) as the effect index with 95% confidence interval. The *I*^2^ statistic will be used to assess levels of the heterogeneity, when *I*^2^ < 50%, the fixed-effect model can be used for analysis, otherwise, the random-effect model will be used.

### 2.7. Sensitivity analysis and subgroup analysis

We will consider the subgroup analysis intervention of the experimental group. In addition, through sensitivity analysis assess the source of heterogeneity, by excluding low-quality studies, or by excluding one of the included studies in turn, if there is no significant change in the heterogeneity, the results are robust, otherwise, the excluded study may be the heterogeneous originate.

### 2.8. Publication bias

In this study, less than 10 RCTs will use funnel plots to evaluate publication bias, or else, Egger regression test will be used. Reporting bias, if any, will be assessed by comparing the study results with its protocol.

## 3. Discussion

The use of medication for KOA is strongly supported in stepped knee preservation. The traditional TCM compound DGSND has been proved to be effective in history and can help improve the symptoms of patients, but there has been no high-level clinical evidence-based basis to convince scholars. The purpose of this study is to solve this problem and to provide evidence-based basis for clinical decision-making.

## Author contributions

**Conceptualization:** Kemeng Xiang, Xing Zhou.

**Data curation:** Xing Zhou.

**Formal analysis:** Xing Zhou.

**Investigation:** Guang Yang, Hanting Xia, Kemeng Xiang, Xing Zhou, Yanbo Wang.

**Methodology:** Hanting Xia, Ke-Meng Xiang, Yanbo Wang.

**Resources:** Guang Yang.

**Software:** Hanting Xia, Ke-Meng Xiang.

**Supervision:** Jinlei Li, Rujie Zhuang.

**Validation:** Jinlei Li.

**Writing – original draft:** Ke-Meng Xiang.

**Writing – review & editing:** Rujie Zhuang, Xing Zhou.
